# Supplementary figures and images for: Using viral sequence diversity to estimate time of HIV infection in infants
Source: PLoS Pathog. 2023 Dec 20;19(12):e1011861. doi: 10.1371/journal.ppat.1011861 (PMC10732395; doi:10.1371/journal.ppat.1011861)

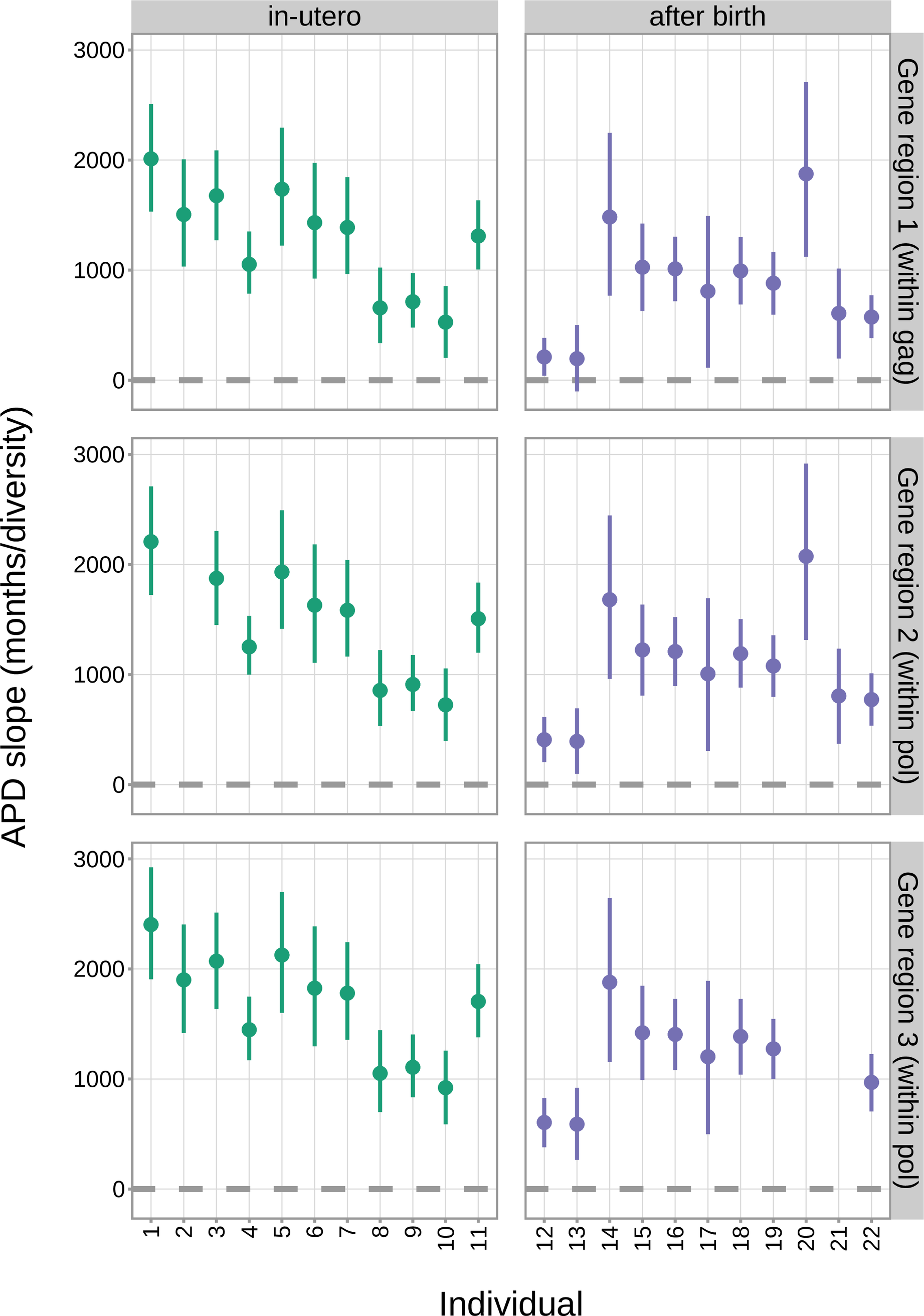

Supplement: S1 Fig — The inferred distribution of APD slope for each individual and gene region in the training data set obtained using Markov Chain Monte Carlo sampling from the Bayesian hierarchical model (described above) is shown. The median slope values are plotted, along with the 89% credible interval for the parameter distribution. An APD slope equal to zero is shown by the horizontal dashed line. APD slopes are shown in units months/diversity–as such, a higher rate indicates that diversity is accumulating slower with time. (TIF) [file ppat.1011861.s001.tif]

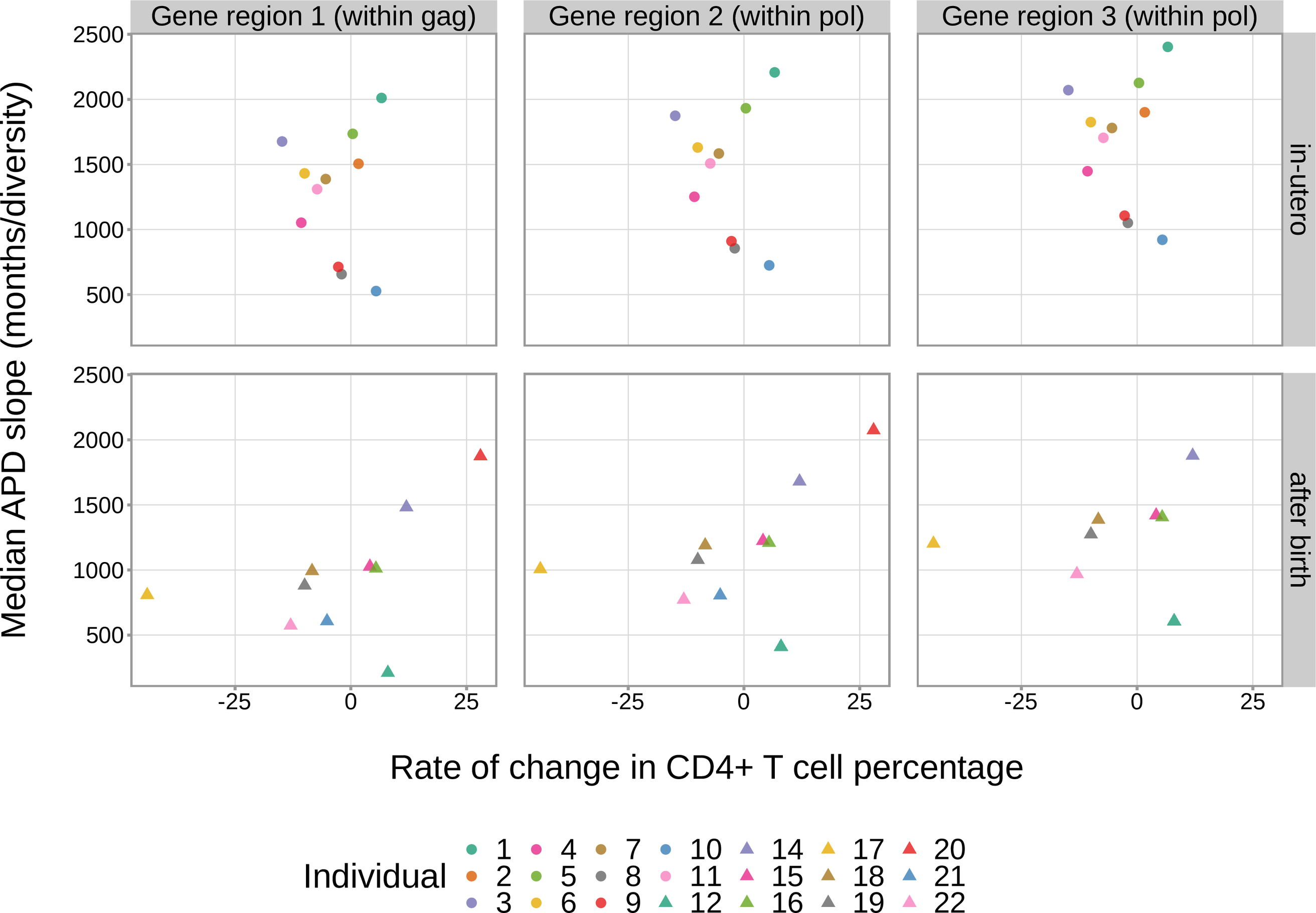

Supplement: S2 Fig — The inferred median APD slope for each individual and gene region from the infant-trained hierarchical model is shown as a function of the rate of CD4+ T cell percentage decline for each individual. (TIF) [file ppat.1011861.s002.tif]

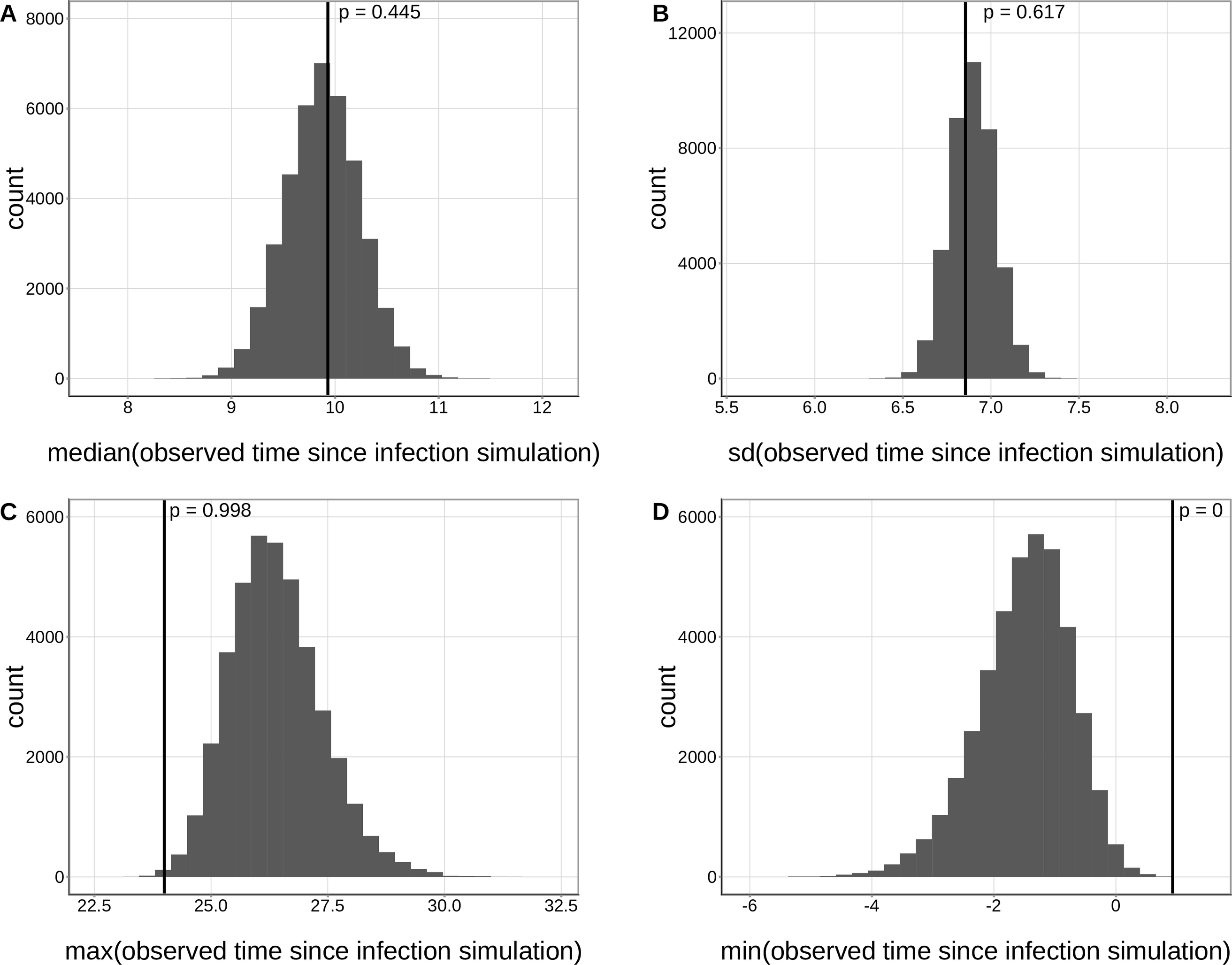

Supplement: S3 Fig — The (A) median, (B) standard deviation, (C) maximum, and (D) maximum test statistics for the distribution of true observed time since infection measures are shown by the vertical black line. The p-values from comparing each test statistic computed for the true observed time since infection measures to the observed time since infection measures simulated from the model are also shown. The unit for each test statistic is months. (TIF) [file ppat.1011861.s003.tif]

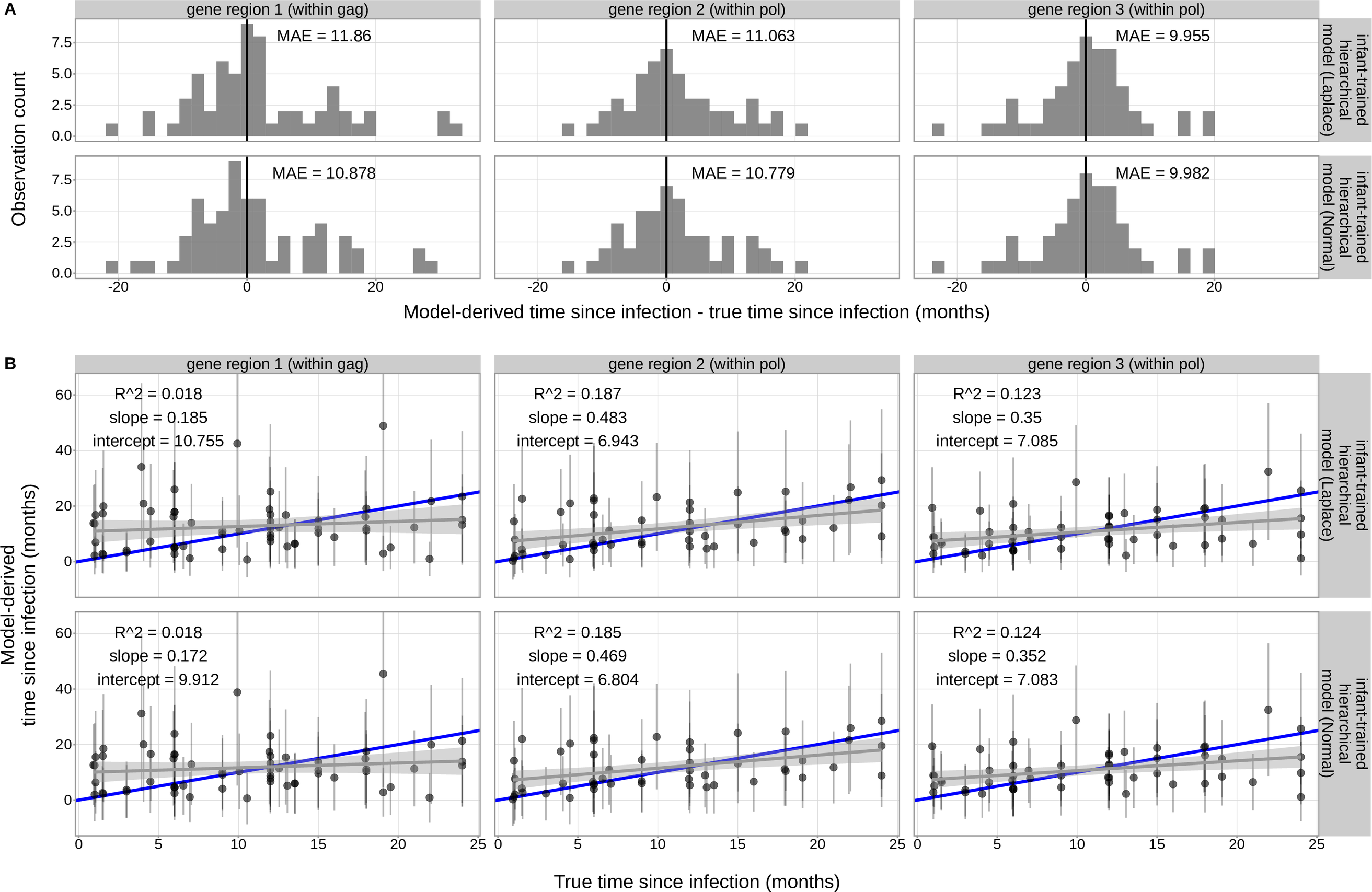

Supplement: S4 Fig — Leave-one-out-cross-validation was used to calculate the estimated time since infection for each individual. (A) Drawing observations from a Laplace distribution instead did not improve prediction accuracy. The difference between true time since infection and model-derived time since infection is shown on the x-axis. A difference of zero is shown by the black vertical line. (B) Comparison of true time since infection and model-derived time since infection for both models using APD measures sampled from gene region 3. The 89% credible interval is shown as a vertical black line for each estimate. The blue line represents the model-derived time since infection = true time since infection line. The gray line shows the line of best fit. (TIF) [file ppat.1011861.s004.tif]

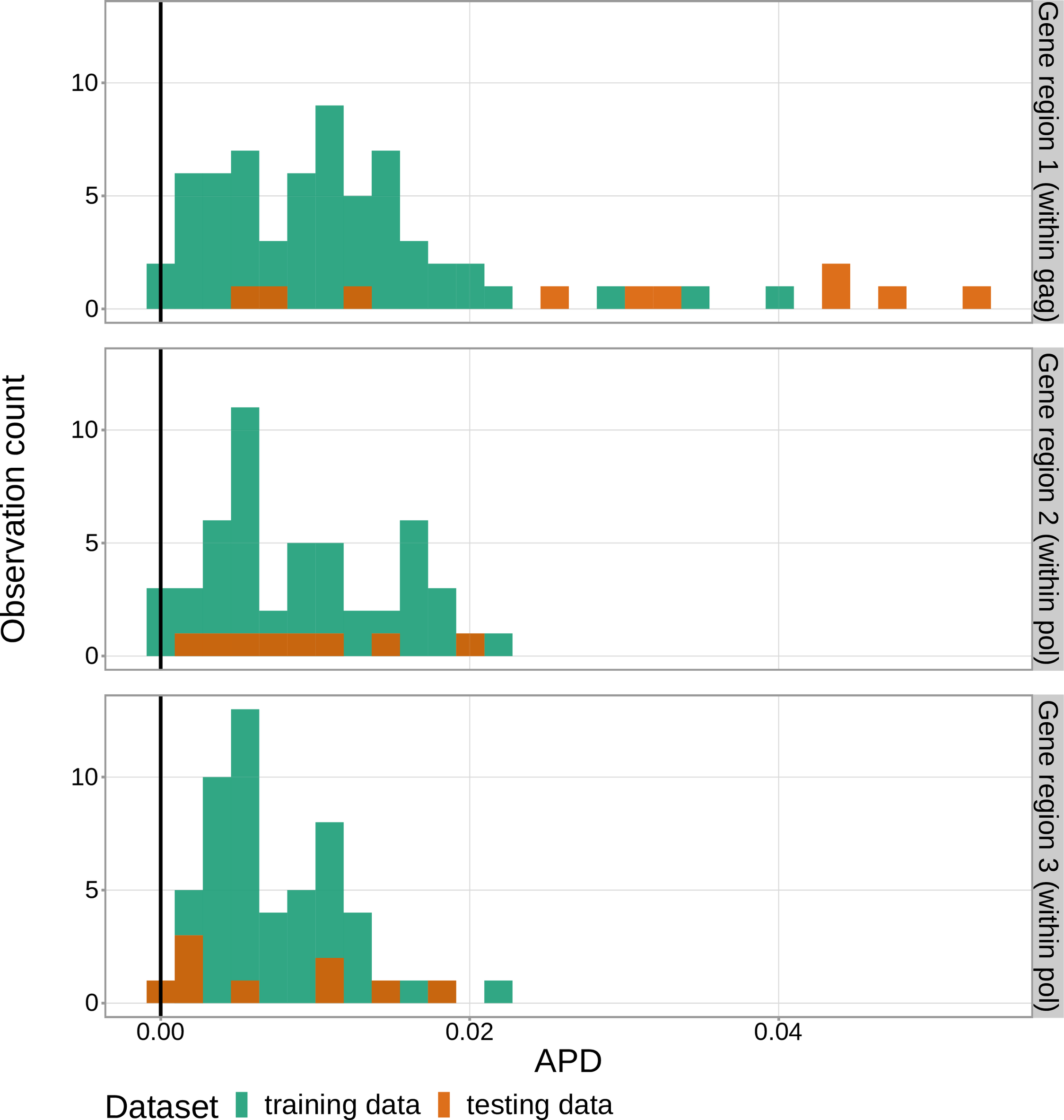

Supplement: S5 Fig — The testing data set has much higher APD measures within gene-region 1 compared to the training data set. (TIF) [file ppat.1011861.s005.tif]

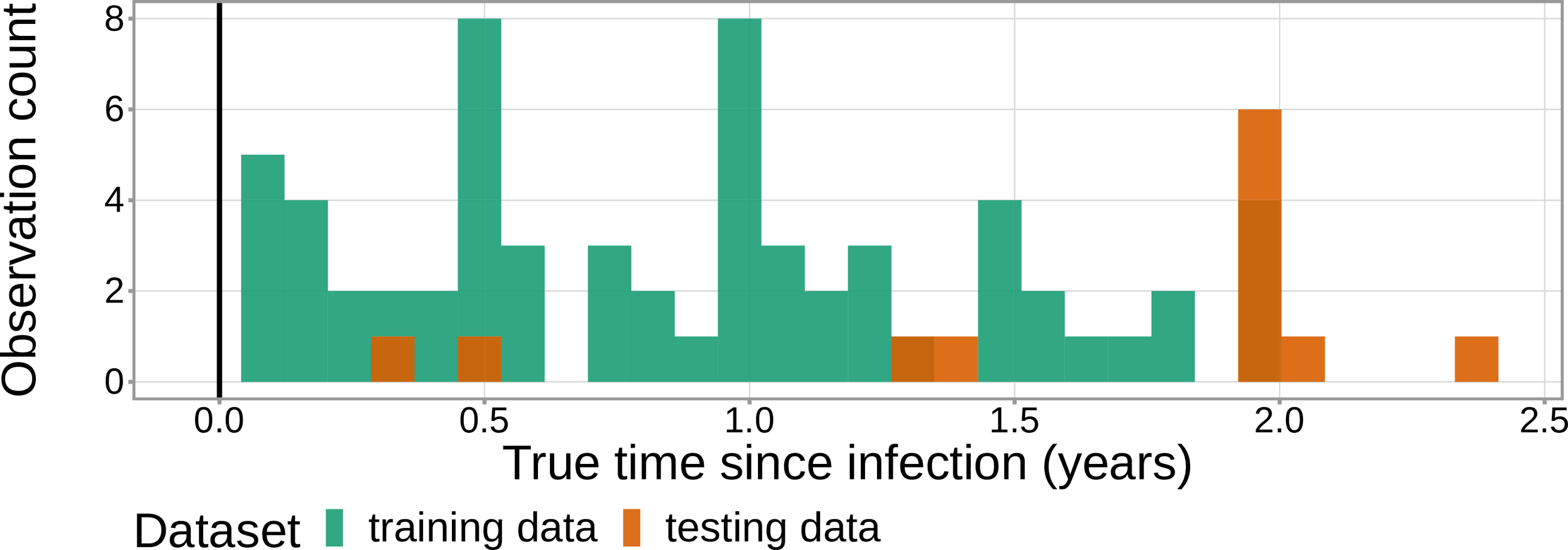

Supplement: S6 Fig — The testing data set has much higher time measures compared to the training data set. (TIF) [file ppat.1011861.s006.tif]
