# Supplementary material for: Using viral sequence diversity to estimate time of HIV infection in infants
Source: PLoS Pathog. 2023 Dec 20;19(12):e1011861. doi: 10.1371/journal.ppat.1011861 (PMC10732395; doi:10.1371/journal.ppat.1011861)
Supplement: S1 Table — (PDF) [file ppat.1011861.s007.pdf]

Bayes factor value	Interpretation
> 100	Extreme evidence for the *infant-trained hierarchical model*
30-100	Very strong evidence for the *infant-trained hierarchical model*
10-30	Strong evidence for the *infant-trained hierarchical model*
3-10	Moderate evidence for the *infant-trained hierarchical model*
1-3	Anecdotal evidence for the *infant-trained hierarchical model*
1	Equal evidence for the *infant-trained hierarchical model* and the alternative model
1/3-1	Anecdotal evidence for the alternative model
1/10-1/3	Moderate evidence for the alternative model
1/30-1/10	Strong evidence for the alternative model
1/100-1/30	Very strong evidence for the alternative model
< 1/100	Extreme evidence for the alternative model
