# Supplementary material for: Using viral sequence diversity to estimate time of HIV infection in infants
Source: PLoS Pathog. 2023 Dec 20;19(12):e1011861. doi: 10.1371/journal.ppat.1011861 (PMC10732395; doi:10.1371/journal.ppat.1011861)
Supplement: S2 Table — (PDF) [file ppat.1011861.s008.pdf]

Region	Gene	Primer	Sequence (5' → 3')	HXB2 location
1	*gag*	Reverse Transcription	GGAGTTCAGACGTGTGCTCTTCCGATCTNNNNNNNNACTGTATCATCTGCTCCTGTRTCT	2324 ⇐ 2383
		Forward	GCGAGAGCGTCAGTATTAAGCGG	796 ⇒ 818
		Reverse	TGCCAAAGAGTGATYTGAGGG	2252 ⇐ 2272
2	5' *pol*	Reverse Transcription	CTRTTAGCTGCCCCATCTACATAG	3869 ⇐ 3892
		Forward	AAATTGCAGGGCYCCTAG	1998 ⇒ 2015
		Reverse	ACAAACTCCCAYTCAGGAATCCA	3777 ⇐ 3799
3	3' *pol*	Reverse Transcription	GGGATGTGTACTTCTGAACTTAYTYTTGG	5185 ⇐ 5213
		Forward (outer)	CACACTAATGATGTAARACARTTAACAG	3630 ⇒ 3657
		Reverse (outer)	GGGATGTGTACTTCTGAACTTAYTYTTGG	5185 ⇐ 5213
		Forward (inner)	GAAAGCATAGTRATATGGGGAAA	3681 ⇒ 3703
		Reverse (inner)	CACCTGCCATCTGTTTTCCATA	5040 ⇐ 5061
