# Supplementary material for: Using viral sequence diversity to estimate time of HIV infection in infants
Source: PLoS Pathog. 2023 Dec 20;19(12):e1011861. doi: 10.1371/journal.ppat.1011861 (PMC10732395; doi:10.1371/journal.ppat.1011861)
Supplement: Attachment — Submitted filename: infection-timing_response.pdf [file ppat.1011861.s009.pdf]

Thank you very much for submitting your manuscript "Using viral sequence diversity to estimate time of HIV infection in infants" for consideration at PLOS Pathogens. As with all papers reviewed by the journal, your manuscript was reviewed by members of the editorial board and by several independent reviewers. In light of the reviews (below this email), we would like to invite the resubmission of a significantly-revised version that takes into account the reviewers' comments.

Part I - Summary

Reviewer #1: Russell and co-authors introduce a Bayesian hierarchical model for estimating timing of infection in infants. They show that training their model on the diversity of viral sequences sampled in utero and postpartum more accurately predicts infection timing than training on sequences sampled from adults (or linear modeling trained on infant sequences). Using this method, they find considerable inter-individual and gene region variation, but overall that pol diversifies faster in infants than adults, irrespective of SPVL or CD4+ decline. The authors have done well to develop a method that takes advantage of their unique dataset and the results are promising for its further use. I have a few outstanding questions regarding how the method was tested and how it will be more widely implemented. -Eric Lewitus

Reviewer #2: In their manuscript, 'Using viral sequence diversity to estimate time of HIV infection in infants', estimated rates of viral diversification in 22 Kenyan children and determined that this rate was three times higher than what is seen in infected adults. In this population the diversification rate did vary by individual, route of infection (during delivery vs. breast feeding) and by viral gene region analyzed. However, set point viral load or rate of CD4 decline did not seem to be associated with differences in viral diversification rates. The authors used a Bayesian hierarchical model trained on either the infant data or existing adult data to determine the best fit to the data from the validation cohort of 15 different infants. They found that the model trained with the data from the Kenyan infants fit the validation model best. Unfortunately, there is too much subject-to-subject variability to differentiate infants who were infected in-utero vs. after birth. Overall, this is a well written and thorough investigation. The figures and tables are clear and appropriate. There are some issues to address.

Thank you for your insightful and constructive comments, which have improved our paper. We are especially grateful for your suggestion to compare our results to a phylogenetic molecular dating approach using BEAST and for your question regarding how the results from our work may impact other studies.

We have summarized the changes relevant to each essential revision here and provided a "diff" file that highlights the differences from the first version.

Part II – Major Issues: Key Experiments Required for Acceptance

Reviewer #1: The state-of-the-art for inferring timing of infection is phylogenetic molecular dating. I think the utility of the authors' method would need to be shown to be superior in ease of implementation and at least comparable in results to, for example, inferences of timing of infection drawn from BEAST analyses. A comparison to Dearlove et al., PLoS Comp Bio, 2021 (or another relevant analysis) would suffice. If Bayesian phylogenetic analysis for molecular dating is not appropriate for their question (or infant sequences), then it would be helpful for the authors to explain why.

Thank you for this suggestion! We agree that evaluating our methods alongside a phylogenetic-based molecular dating approach, particularly a BEAST analysis, would have been an ideal comparison if we had access to single genome sequencing data or long sequencing reads that spanned the entirety of each gene region of interest. Unfortunately, the data we have generated for this study are similar to those used in a previously-published model for adult-specific HIV infection timing (see Puller, et. al PLoS Comp Bio 2017) and consist of short, unlinked sequencing reads that do not span the entirety of any gene region. While these data are well-suited for estimating sequence diversity and evaluating infection timing methods based on these sequence diversity measures, they cannot be used for a BEAST analysis. We have added a section to the Discussion (lines 496-500) to acknowledge this shortcoming as follows:

"Here we developed and evaluated methods for estimating infection timing that are suitable for short, unlinked sequencing reads, a data type used in some adult-specific models [1]. Other types of sequencing data enable additional infection timing methodologies. For example, given single genome sequence data, one can use BEAST, a Bayesian phylogenetic software, to conduct a Bayesian phylogenetic analysis for molecular dating. This approach has been previously shown to accurately estimate infection timing of adult HIV infections [43]."

Timing of infection is inferred in years. Given that the individuals are infants, which gives a small window for timing of infection, and that MAE is around 0.5 years, is this enough resolution? What questions regarding timing of infection do the authors imagine would be answered at this resolution? As results are given in fractions of years (e.g., 1.68), it would be useful to report results in months. Otherwise, please explain why years were chosen as units.

We initially chose years as the unit of time since infection for our models to align with adult-specific models published by other groups. However, we agree with this suggestion and have converted the units of time since infection to months throughout the manuscript (for example, see lines 140, 158, 170, etc.). We have also changed the units of time since infection within all relevant figures (e.g. Figure 1, Figure 2, Figure 3, etc.).

However, in order to keep the time since infection units within our modeling code consistent with existing adult-specific methods, we have chosen to continue using years as the time since

infection unit for our modeling. If users would like to convert the units of their modeling results from years to months, this can be easily done after inferences are made.

Reviewer #2: (No Response)

Part III – Minor Issues: Editorial and Data Presentation Modifications

Reviewer #1: Line 145: The studies cited supporting that "the majority of infants are infected with only a single viral variant" are quite limited (3-10 mother-infant pairs) and in one study (Wu et al., 2006) 2/8 infant infections appear to have been established by multiple founder variants, which is on par with adults. As deep sequencing is showing that rare founder variants are more common than previously thought, how would this affect inference with the Bayesian hierarchical model? Can it be adapted to infer timing in multi-founder infections? If not, I think it is worth saying that the method is only applicable to infections established with a single variant.

This is a good point and we have added the following section within the Discussion (lines 519-521) to highlight this modeling limitation:

"Next, because our infection timing models were formulated using the assumption that the majority of infants are infected with only a single viral variant [23–27], they may produce inaccurate time since infection estimates for individuals with multi-founder infections."

While it is technically feasible to modify our current Bayesian hierarchical model to handle both single and multi-founder infections (e.g. using a unique slope and intercept for each infection type), doing so would require knowledge of the infection type when applying the model to individuals with unknown infection timing. However, because this information may not be available when using diversity measures sampled from a single time point, we have opted to limit our modeling to the assumption of single variant infections.

Line 157: Considering that certain sites will always be conserved, wouldn't it make more sense to compute the APD backwards in time to when diversity was zero? A result of 105 years — which if I understand correctly means that it would take 105 years for all sites between two sequences to be mismatched — is difficult to conceptualize. Perhaps the authors could explain further why they chose to look at the rate of diversity accumulation forwards in time.

We have designed the model such that it could use APD measures to predict time since infection using a linear-regression-style approach. Consequently, the inferred APD slopes from this model are in units of time per diversity, aligning with this predictive set-up. A similar approach was taken in previous studies estimating rates of viral diversification in adult cohorts (e.g. Puller et. al. PLoS Comput Biol. 2017 and Carlisle et. al. Journal of Infectious Diseases.

2019). Given that the time required for diversity to equal zero in “backwards time” will depend on the sequence, it is not clear how we would set up a regression model using backwards time.

With that being said, we agree that it is difficult to interpret a given APD slope measure directly. Instead, we suggest considering the inverse of this quantity which offers a more interpretable measure. We have expanded the following section in the Results (lines 156-163) to further explain this idea:

“Each viral sequence diversity (APD) measure represents the probability that two randomly drawn sequences have different nucleotides at a specified position, averaged over all positions, and the APD slope (i.e. rate of APD accumulation measured in units of months per diversity) represents the rate at which the APD measure increases. Because mutations will saturate within a sequence over time, APD slope should not be interpreted as the time required for all sites between two sequences to be mismatched. Instead, we suggest considering the inverse of APD slope, measured in units of diversity per month, which can be interpreted as the rate at which mismatches between two sequences accumulate.”

Lines 225-237: If the authors “did not use this alternative model for downstream analyses”, why was it presented? Could the authors explain what this alternative model brings to the manuscript?

One of the main goals of our work was to gain new biological insights by exploring the axes under which APD slopes varied. The alternative model introduced on lines 234-235 contained a mode-of-infection-specific slope-modifying term which allowed us to evaluate whether APD slopes varied by mode of infection using a Bayes Factor test. Despite the Bayes Factor test providing extremely strong evidence in favor of this alternative model, we could not include the mode-of-infection-specific slope-modifying term in the final model formulation since we did not expect to have knowledge of mode of infection when applying the final model to individuals with unknown infection timing. As such, while this alternative model was not used for downstream analyses, it still allowed us to explore the interesting biological question of whether APD slopes varied by mode of infection. We have added the following section in the Results (lines 246-249) to clarify this:

“Regardless of whether the variation in APD slopes by mode of infection represents a true biological signal, we chose not to use the alternative model that includes a mode-of-infection-specific slope-modifying term for downstream analyses. This decision was made because we do not expect to have knowledge of the mode of infection when applying our final model to individuals with unknown infection timing.”

Lines 427-429: If individuals 12 and 13 did not test positive at birth, why would they have elevated rates compared to individuals infected in utero and test positive at birth? Does the difference between in utero and postpartum rates disappear if these individuals are removed?

Thanks for raising this question. We found that postpartum-infected individuals had a higher rate of viral diversification than in-utero-infected individuals. Individuals 12 and 13 (both of whom were postpartum-infected) had relatively higher rates of viral diversification than other postpartum infected individuals. When we removed these two individuals and repeated our analysis exploring whether APD slopes varied by timing of infection, we found that there was no longer a substantial difference in APD slopes between the two groups. To highlight this, we have added the following section to the Results (lines 242-246):

"A reviewer suggested that this signal was driven by the two postpartum-infected individuals (individuals 12 and 13) who had relatively higher rates of viral diversification compared to the other postpartum-infected individuals. Indeed, we found that APD slope did not vary substantially with mode of infection (BF = 2.203) when these two individuals were excluded from the analysis."

And we have added the following section to the Discussion (lines 447-448):

"In fact, when we removed these individuals from our analysis, we no longer found a difference in viral diversification rates between the two groups."

Lines 466-477: Could the model instead be fit with a non-linear model? Or something responsive to Ne?

This is a great point! We would have loved to fit a more complex model. However, given that we only have sequence diversity sampled for 2-3 time points per individual in these data, we believe that a non-linear and/or Ne-responsive model would likely be overparameterized. We have expanded the following sentence within the Discussion section (lines 492-494) to suggest these as possible models if data consisting of more frequent diversity sampling were available:

"Further work consisting of more frequent diversity sampling during early infection will be required to explore these relationships and formulate appropriate regression models (i.e. nonlinear models, models responsive to viral population size, etc.)."

Line 481: Were there any differences in mothers who received ART during pregnancy?

This is a good question. The majority of the mothers of the infants included in the training cohort received antiretroviral therapy during their pregnancies (e.g. 19 of 22 mothers), all of which received only a short-course of zidovudine (AZT) to reduce the risk of mother to child transmission. Because there were only three mothers who did not receive antiretroviral therapy during pregnancy, we would have had limited statistical power to detect whether antiretroviral therapy during pregnancy affected infant rates of viral diversification using our model. As such, we chose not to do this analysis for the study. Instead, we expanded the following section (lines 505-512) within the Discussion to explain this:

“Next, while all of the infants included in this study were naive to antiretroviral therapy for the duration of their monitoring, many of their mothers received a short course of zidovudine (AZT) during pregnancy, which was standard of care in Kenya at the time of cohort enrollment to reduce the risk of mother-to-child transmission. In fact, for the infants included in the training cohort, only 3 out of 22 mothers did not receive short-course AZT during their pregnancies. While it is possible that this lack of treatment could have influenced the rates of viral diversification for the infants born to these mothers, we did not have the statistical power to explore this relationship due to the small number without AZT.”

Reviewer #2: 1) The last sentence of the introduction, on line 61, the authors state that, “These findings hold promise for developing infant-specific treatment approaches and preventive measures.” What are the possible treatment procedures and prevention measures that would use the findings presented in the manuscript? This should be addressed in the discussion.

We agree that it is challenging to provide concrete examples of infant-specific treatment approaches and preventive measures based on the differences we show in rates of HIV diversification between pediatric and adult HIV infections, and so we have chosen to remove this sentence from the manuscript (e.g. lines 60-61) and replace it with the following section which highlights the broader implications of our work:

“These findings also highlight the importance of considering these differences when developing methodologies for future studies related to HIV infection timing across different age groups, as failing to do so may result in incorrect conclusions regarding the timing of pediatric infections.”

2) On line 481, the authors state that some of the mothers were on ART at the time of birth. Why was exposure not added to the model to see if it mattered?

We appreciate your question, and it’s worth noting that Reviewer 1 had a similar suggestion. The training cohort in our study primarily consisted of infants born to mothers who received short-course AZT during their pregnancies (e.g. 19 out of 22 mothers). Due to the limited number of mothers (only three) who did not receive AZT during pregnancy, conducting an analysis to determine the impact of this treatment on infant viral diversification rates using our model would have had limited statistical power. Because of this, we chose not to do this analysis for the study and we expanded the following section (lines 505-512) within the Discussion to explain this:

“Next, while all of the infants included in this study were naive to antiretroviral therapy for the duration of their monitoring, many of their mothers received a short course of zidovudine (AZT) during pregnancy, which was standard of care in Kenya at the time of cohort enrollment to reduce the risk of mother-to-child transmission. In fact, for the infants included in the training cohort, only 3 out of 22 mothers did not receive short-course AZT during their pregnancies.

While it is possible that this lack of treatment could have influenced the rates of viral diversification for the infants born to these mothers, we did not have the statistical power to explore this relationship due to the small number without AZT.”

3) Line 425 states, “In fact, we found that only one individual (individual 485) had a decrease in viral diversity over time across all gene regions.” However, only individuals numbered 1 to 21 are presented. Based on Figure 1 panel A, I believe the authors are referring to individual #7.

Thank you for bringing this error to our attention. We have corrected the numbering of individual 485 to 10 in the text.

4) It is surprising that the rates of diversification for HIV are three-fold faster in infants than in adults. This is especially true given the infant's undeveloped immune system at the time of infection. In adults whose immune systems are severely compromised (like end stage AIDS), the rates of diversification are less than when their immune system is intact (see Shankarappa J Virol 1999 for example). These individuals also have very high viral loads, similar to that seen in infants. Adaptation to a new host could be the reason for the increased viral evolution that is observed. This would be supported by the author’s observation that the rate of diversification decreases after the first year.

This is a fascinating suggestion! We have expanded the following sentence within the Discussion section (lines 487-489) to include the hypothesis that adaptation to a new host could be the reason for increased viral replication during early infection:

“This may be possible if, for example, rapidly increasing viral load levels during very early infection result in relatively higher rates of viral replication/diversification, perhaps as a result of viral adaptation to a new human host, compared to when set-point viral load levels are established later on.”

5) How would the impact of accelerated HIV sequence evolution in infants impact other studies? For example, the outbreak in children attending the Al-Fateh Hospital in Benghazi, Libya (see de Oliveira Nature 2006), if a different rate of diversification for the infants was used would that implicate the foreign medical staff in infecting the infants?

Thank you for your question. We have added the following section to the Discussion section (lines 429-433) to highlight that accelerated HIV sequence diversification in infants could impact other studies:

“This finding suggests that viral diversity accumulates much faster during pediatric infection relative to adult infection and highlights the importance of considering these differences when developing methodologies for future studies related to HIV infection timing across different age groups. Depending on the study, failure to appropriately account for accelerated rates of HIV sequence evolution in infants could result in erroneous conclusions regarding the timing and/or source of pediatric infections.”

Regarding the mentioned example of the study involving the HIV outbreak in children attending the Al-Fateh Hospital study in Benghazi, Libya (presented in de Oliveira et. al. Nature 2006): The authors estimated the HIV evolutionary rate using reference strain sequences (i.e. from an HIV sequence database) that were not necessarily sampled from children. They encoded this rate as a prior probability distribution for their modeling, despite the downstream analysis involving sequences from children. While this choice could have influenced the conclusions of this study, it is challenging to assess the impact of a potentially misspecified prior on the outcome of a Bayesian phylogenetic analysis.
